# Estimation of the Seroconversion Duration of HIV-1 Antibodies in Individuals With Recent Infection in China

**DOI:** 10.3389/fmicb.2019.01322

**Published:** 2019-06-12

**Authors:** Wen-Hua Kong, Pan Liu, Li Tang, Ze-Rong Zhu, Peng Xiao, Jian-Bo Zhan, Xia Wang, Wang Zhou, Man-Qing Liu

**Affiliations:** ^1^Department of Pathogen, Wuhan Centers for Disease Prevention and Control, Wuhan, China; ^2^Hubei Provincial Center for Disease Control and Prevention, Wuhan, China

**Keywords:** seroconversion, HIV/AIDS, Western blot bands, viral marker, recent HIV-1 infection

## Abstract

The identification of recent HIV-1 infection is clinically important for the effective treatment and prevention of transmission. However, the window period for seroconversion with respect to various HIV-1 antibodies is not well characterized. In addition, the routine HIV testing algorithms are not particularly appropriate for the identification of recent HIV-1 infection. In this study, we enrolled individuals who showed seroconversion from negative Western blot (WB) or indeterminate WB results and analyzed the window periods for appearance of HIV-1 antibodies. A total of 10,934 individuals with suspected HIV infection were tested by Wuhan CDC between 2012 and 2017; of these, 40 individuals with initial negative WB and 102 individuals with initial indeterminate WB who showed positive WB results within 100 days were included in the analysis. The mean time for seroconversion was 43.90 (95% confidence interval [CI]: 37.30–50.50) days and 42.15 (95% CI: 37.99–46.30) days, respectively. The time duration for p31 seroconversion among people with negative WB and indeterminate WB was 58.11 (95% CI, 44.30–71.92) days and 51.91 (95% CI, 44.55–59.28) days, respectively, both of which were significantly longer (*p* = 0.0169) than those in people without p31 seroconversion. A similar difference was observed with respect to p66 seroconversion, with a window time of 53.53 (95% CI, 43.54–63.52) days and 47.87 (95% CI, 43.16–52.57) days among people with negative WB and indeterminate WB, respectively. These data suggest that HIV-1 antibody p66, like p31, may serve as a potential serological marker for distinguishing Fiebig stage V and stage VI at day 70 post-infection.

## Introduction

HIV/AIDS continues to be a major global public health crisis with wide social ramifications. In the year 2017, an estimated 1.8 million new cases of HIV infection and 0.94 million HIV-related deaths were reported across the world (World Health Organization [WHO], 2017). Given the UNAIDS 90-90-90 target to end AIDS in 2030 (Brostrom et al., 2014), expansion of the access to HIV testing and improvement in screening algorithms is a key imperative, and the increasing detection of recent HIV-1 infection in clinical practice has posed to be a major challenge (Schupbach et al., 2007; Cohen et al., 2010; Branson and Stekler, 2012; Liu et al., 2016; Bottone and Bartlett, 2017; Ning et al., 2018; Toussova et al., 2018). Recent HIV-1 infection, also known as early HIV-1 infection or primary HIV-1 infection, is usually defined as detectable HIV-1 RNA or p24 antigen in serum or plasma in the setting of negative or indeterminate result of HIV-1 antibody test including Western blot (WB) (Pilcher et al., 2010). Recent HIV-1 infection can be confirmed by subsequent HIV antibody seroconversion. The identification of recent HIV-1 infection is extremely useful for antiretroviral treatment and for pathogenetic and epidemiologic studies (Cohen et al., 2010; Hecht et al., 2011).

According to the staging of recent HIV-1 infection by Fiebig et al. (2003) (Cohen et al., 2011b), the progression of HIV-1 infection can be divided into six separate stages based on the results of sequential laboratory tests. Of these, the fourth-generation assays for detection of HIV-1 and HIV-2 antibodies (Ab) and HIV-1 p24 antigen (Ag) can be used to detect HIV infection after the eclipse phase and stage I, while results of WB are negative in stage II and III, and indeterminate in stage IV. In stage V (day 31–100 post-infection), HIV-1 antibodies that bind to fixed viral proteins would result in WB reactive, while p31 band remains non-reactive (Fiebig et al., 2003; Cohen et al., 2010, 2011b). Several studies have found that the pattern of WB bands is associated with recent HIV-1 infection (Schupbach et al., 2007, 2011; Wang et al., 2013) and disease progression (Garland et al., 1996). For example, p31 can be used as a viral marker to distinguish Fiebig stage V and stage VI (Cohen et al., 2011b). In a study by Sudha et al. (2006), p31 was the most frequently missing band, followed by p55, p66, p51, and gp41. The emergence of HIV-1 WB bands at different time-points during the early infection may reflect the interaction between the virus and the host. However, data pertaining to seroconversion duration for each HIV-1 antibody are limited and the relation of various WB bands with disease progression is not well characterized. In order to further understand the window period for appearance of HIV-1 antibodies, we retrospectively analyzed the seroconversion time of individuals with recent HIV-1 infection in Wuhan, China.

## Materials and Methods

### Diagnosis of HIV-1 Infection

From 2012 to 2017, plasma or serum specimens were initially screened for HIV antigen and/or antibody at local Centers for Disease Prevention and Control (CDCs), hospitals, blood centers, and other health screening centers in Wuhan, China. The specimens that were reactive on the initial assay were sent to Wuhan CDC for confirmation. All HIV-suspected samples were further screened with two 4th generation Ag/Ab HIV-1/2 enzyme immunoassays. Samples with at least one reactive result in the subsequent screening were confirmed with HIV BLOT 2.2 WB kit (Mp Biomedicals Asia Pacific Pte. Ltd., Singapore). As recommended by the manufacturer of WB kit, specimens with two envelope proteins (gp160/gp41 and gp120) plus one of the core proteins (p17, p24, and p55) or one of the enzyme proteins (p31, p51, and p66) were defined as HIV-1 antibody positive; specimens that exhibited HIV-1 bands (except p17) yet did not qualify for the minimum criteria for positive result were defined as indeterminate (Linley et al., 2013; Moon et al., 2015; Liu et al., 2016). The WB bands for all specimens were visually verified by at least two experts independently. As per the National Guidelines for Detection of HIV/AIDS in China (2015 Version), all subjects with indeterminate HIV-1 WB results were asked to undergo re-testing after 2–4 weeks. In addition, patients with suspected acute HIV-1 infection who showed negative HIV-1 WB result were also recommended to undergo repeat testing after 2–4 weeks (Liu et al., 2016).

### Data Collection and Analysis

All individuals with suspected HIV-1 infection were registered in the Wuhan HIV Management Database. Subjects that underwent repeat testing were retrospectively tracked using the unique identification number, especially individuals who showed positive WB results after initial negative or indeterminate WB test result. Seroconversion duration of HIV-1 antibody was estimated based on the interval between two sampling dates. Individuals with duration ≥100 days were excluded in order to eliminate the possibility of multiple exposures.

### Statistical Analysis

Where appropriate, data are expressed as mean ± standard deviation (SD). Statistical analyses were performed with GraphPad Prism (Graphpad Software Inc., San Diego, CA, United States). Between-group differences with respect to categorical variables were assessed using the Chi-Squared test; those with respect to continuous variables were assessed using the Student’s *t*-test. *P*-values <0.05 were considered indicative of statistical significance.

## Results

### Basic Information

**FIGURE 1:**
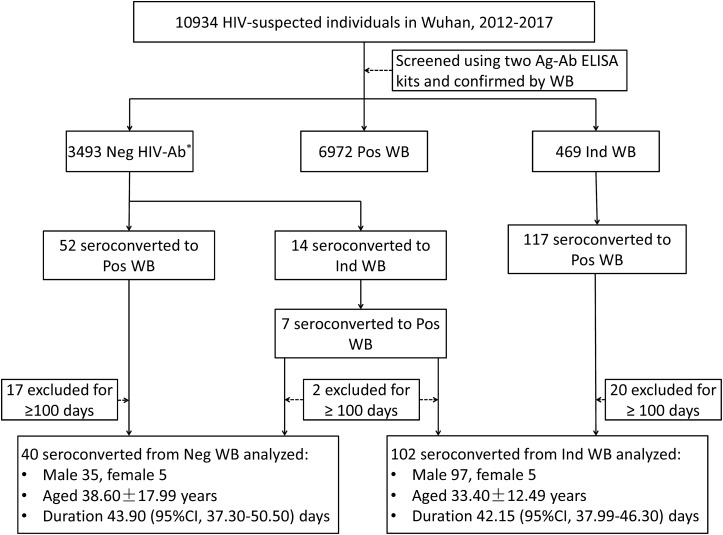
Schematic illustration of the study design and patient selection criteria. During the study reference period (2012–2017), 10934 individuals with suspected HIV infection were screened with two Ag-Ab ELISA kits and confirmed by Western blot in Wuhan CDC. Of these, 59 patients and 124 patients with negative WB and indeterminate WB, respectively, showed seroconversion (including 7 patients who initially seroconverted from negative to indeterminate WB and finally to positive WB). After exclusion of patients who seroconverted after ≥100 days, 40 patients with negative WB and 102 patients with indeterminate WB who showed seroconversion were included in the analysis. WB, Western blot; Neg, negative; Ind, indeterminate; Pos, positive; CI, confidence interval. ^∗^Including non-reactive for two ELISA kits and WB.

A total of 10,934 HIV-suspected individuals were screened for HIV-1/2 antibodies during the study reference period (2012–2017). Of these, 6972 patients tested positive for HIV-1 antibody in their first test. Among the patients who initially showed negative or indeterminate WB results, 59 patients and 124 patients have seroconverted to positive WB, respectively. After the exclusion of subjects with seroconversion duration ≥100 days, 40 patients who seroconverted from negative WB and 102 patients who seroconverted from indeterminate WB were included in the analysis of seroconversion time (Figure 1). There were no significant differences between the groups that seroconverted from negative WB and indeterminate WB with respect to sex and age (*p* = 0.1446 and 0.0523, respectively) (Figure 1).

### Time Duration for Seroconversion in Individuals With Negative WB

**FIGURE 2:**
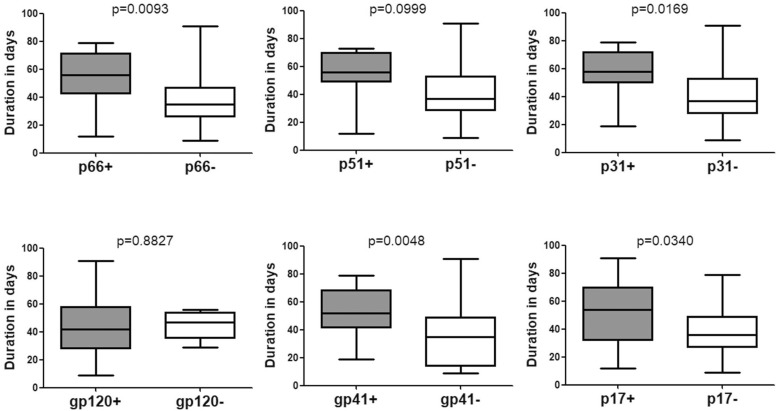
Comparison of the seroconversion duration of different WB bands in HIV-1 positive individuals who seroconverted from negative WB. The time interval (days) between two sampling dates of negative WB and positive WB bands, including p66, p51, p31, gp120, gp41, and p17, were calculated; seroconversion rates for subjects with and without the above bands were compared using the Student’s *t*-test (GraphPad Instat Statistical Software). Thick horizontal bars indicate the median, boxes show quartiles, and whiskers show full range.

The time duration for seroconversion from negative WB to positive WB ranged from 9 to 91 days with a mean duration of 43.90 days (95% confidence interval [CI]: 37.30–50.50) (Table 1 and Figure 1). According to the stages defined by Fiebig et al. (2003) (Cohen et al., 2011b), the mean duration for people who seroconverted from negative WB to Fiebig stage V with positive WB but without p31 was 39.77 (95% CI: 32.53–47.02) days (Figure 2). As the sequential emergence of HIV-1 antibodies is a key indicator of recent HIV-1 infection (Schupbach et al., 2007, 2012; Wang et al., 2013), we further analyzed the window period for each WB band. The mean seroconversion time (from negative WB to positive) for p66, p51, p31, gp120, gp41, and p17 antibodies was 53.53 (95% CI: 43.54–63.52), 52.64 (95% CI: 38.89–66.38), 58.11 (95% CI: 44.30–71.92), 43.71 (95% CI: 36.22–51.21), 52.38 (95% CI: 44.58–60.18), and 51.11 (95% CI: 40.36–61.85) days, respectively. As for p66, p31, gp41 and p17 antibodies, the mean seroconversion duration of patients having these bands in the final WB test were significantly longer (*p* < 0.05) than those of patients who lacked these bands (36.78 [95% CI: 28.62–44.95] days for p66 negative, 39.77 [95% CI: 32.53–47.02] days for p31 negative, 34.53 [95% CI: 24.63–44.42] days for gp41 negative, 37.38 [95% CI: 29.63–45.13] days for p17 negative) (Figure 2).

**Table 1:** Seroconversion duration for HIV-1 antibodies against p31 and p66.

WB bands	Seroconversion from negative WB		Seroconversion from indeterminate WB	
	*n* (%)	Duration in days (95% CI)	*n* (%)	Duration in days (95% CI)
p31+p66−	2 (5.00%)	50	2 (1.96%)	58
p31–p66+	10 (25.00%)	48.70 (35.49, 61.91)	34 (33.33%)	44.29 (38.51, 50.08)
p31+p66+	7 (17.50%)	60.43 (41.91, 78.95)	33 (32.35%)	51.55 (43.97, 59.12)
p31–p66−	21 (52.50%)	35.52 (26.73, 44.31)	33 (32.35%)	29.58 (22.78, 36.38)
Total	40 (100%)	43.90 (37.30, 50.50)	102 (100%)	43.15 (37.99, 46.30)

*WB, Western blot; CI, confidence interval.*

### Time Duration for Seroconversion in Individuals With Indeterminate WB

**FIGURE 3:**
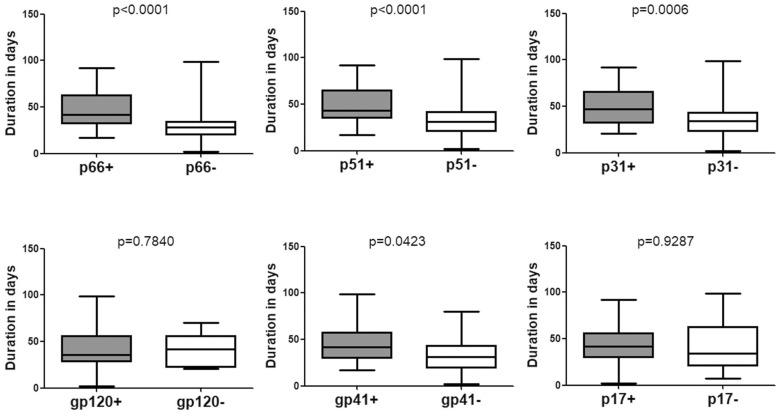
Comparison of the seroconversion duration of different WB bands in HIV-1 positive individuals who seroconverted from indeterminate WB. The time interval (days) between two sampling dates of indeterminate WB and positive WB bands, including p66, p51, p31, gp120, gp41, and p17, were calculated. Seroconversion rates for subjects with and without the above bands were compared using the Student’s *t*-test (GraphPad Instat Statistical Software). Thick horizontal bars indicate the median, boxes show quartiles, and whiskers show full range.

Data pertaining to individuals who seroconverted from indeterminate WB were also analyzed. The mean duration was 42.15 (95% CI: 37.99–46.30) days for seroconverting into positive WB (Table 1 and Figure 1) and 37.04 (95% CI: 32.35–41.74) days for seroconverting into Fiebig stage V. Neither was shorter than the duration for seroconversion in individuals with negative WB, as the Fiebig stages I–IV were relatively brief with an average time of 3–5 days (Fiebig et al., 2003). With respect to single antibodies, there were significant differences between the seroconversion time of individuals with and without p66, p51, or p31 bands in their final WB results (Figure 3). The window periods were 47.87 (95% CI: 43.16–52.57) days for p66, 50.24 (95% CI: 44.68–55.79) days for p51 and 51.91 (95% CI: 44.55–59.28) days for p31, while the mean duration for seroconversion of patients without these bands was 31.20 (95% CI: 24.16–38.24) days, 34.06 (95% CI: 28.57–39.55) days, and 37.04 (95% CI: 32.35–41.74) days, respectively.

**FIGURE 4:**
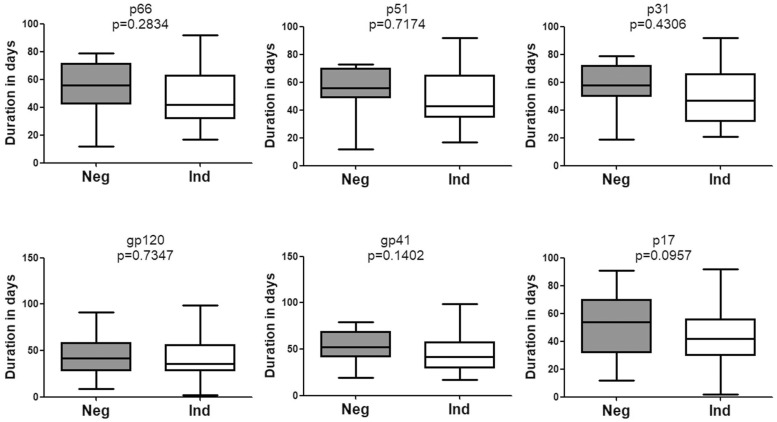
Comparison of the seroconversion for HIV-1 positive individuals who converted from negative WB and indeterminate WB. The time interval (days) between two sampling dates of negative/indeterminate WB and positive WB bands, including p66, p51, p31, gp120, gp41, and p17, were calculated. Seroconversion duration for subjects with above bands who seroconverted from negative and indeterminate WB was compared using the Student’s *t*-test (GraphPad Instat Statistical Software). Thick horizontal bars indicate the median, boxes show quartiles, and whiskers show full range. Neg, negative; Ind, indeterminate.

Of note, no significant difference was observed with respect to the emergence time of WB bands between patients who seroconverted from negative WB and those who seroconverted from indeterminate WB (Figure 4). Besides, seroconversion showed no correlation with the pattern of bands in the first WB test (data not shown).

### Potential Viral Markers

P31 was recognized as a HIV-1 marker for distinguishing Fiebig stage V and stage VI during recent HIV-1 infection (Fiebig et al., 2003; Cohen et al., 2011b; Tuaillon et al., 2017). In this study, we observed a significant difference between the seroconversion duration of patients with and without p31 band (*p* < 0.01), irrespective of the first WB test result (negative or indeterminate) (Figure 2, 3). The cut-off duration for p31 antibody seroconversion from negative WB and indeterminate WB was about 45 (44.30–47.02) days and 43 (41.74–44.55) days, respectively. Interestingly, the p66 antibody, like p31, was found to be a potential viral marker, as its window periods (53.53 [95% CI, 43.54–63.52] days from negative WB and 47.87 [95% CI, 43.16–52.57] days from indeterminate WB) were significantly longer (*p* < 0.01, Figure 2, 3) than that for seroconversion of patients without p66 band (36.78 [95% CI, 28.62–44.95] days from negative WB, 31.20 [95% CI, 24.16–38.24] days from indeterminate WB). The cut-off duration for p66 antibody seroconversion from negative WB and indeterminate WB was about 44 (43.54–44.95) days and 42 (38.24–43.16) days, respectively. No significant difference was found between the seroconversion time for p31 and p66 antibodies; in addition, seroconversion with respect to p31 and p66 antibodies was concordant in the majority of patients, including 70.00% of individuals who seroconverted from negative WB and 64.70% of individuals who seroconverted from indeterminate WB (Table 1). In contrast, the differences of seroconversion time associated with p51, gp41, and p17 antibodies were only observed among some patients: gp41 and p17 among patients who seroconverted from negative WB, while p51 in patients who seroconverted from indeterminate WB. There was no difference associated with gp120 in both groups (*p* = 0.8827 and 0.7840) (Figure 2, 3).

## Discussion

The WB assay detects the HIV-1 antibodies that bind to fixed HIV-1 proteins. Globally, it is the most commonly used method for clinical confirmation of HIV-1 infection (Centers for Disease Control [CDC], 2014; Liu et al., 2016; Kondo et al., 2018) and positive WB result is a prerequisite for antiretroviral therapy and laboratory follow-up including CD4 count and HIV-1 viral load test in many low- and middle-income countries. Although HIV-1 nucleic acid testing (NAT) has been recommended for HIV-1 screening, particularly for individuals with acute HIV-1 infection and late-stage AIDS (Tang et al., 2008; Patel et al., 2012; Centers for Disease Control [CDC], 2014; Liu et al., 2016), it is not commonly used for diagnosis due to its cost and technology threshold. In addition, according to the diagnostic criteria for HIV/AIDS (WS 293-2008) in China (Shao et al., 2008), confirmation of HIV-1 infection by NAT requires two positive results with different sampling times, which prolongs the diagnostic process. However, early diagnosis of HIV-1 infection, especially for recent HIV-1 infection, is very important to prevent the spread of HIV-1 and to facilitate prompt initiation of treatment. Clinicians and patients face challenges frequently to estimate window periods (Taylor et al., 2015) and the duration of HIV seroconversion accurately. In this study, we retrospectively analyzed 102 individuals with recent HIV-1 infection; of these, 40 individuals had originally shown negative WB result and 102 individuals had shown indeterminate WB result. The mean duration for seroconversion was 43.90 (95% CI: 37.30–50.50) days and 42.15 (95% CI: 37.99–46.30) days, respectively. As the Fiebig stage VI is open-ended (Fiebig et al., 2003) and typically difficult to estimate, we analyzed the duration of seroconversion to stage V, a stage marked by positive WB result without p31 band. The mean duration of stage V seroconversion for individuals with negative WB and indeterminate WB was 39.77 (95% CI, 32.53–47.02) days and 37.04 (95% CI, 32.35–41.74) days, respectively; these were significantly shorter than that reported by Fiebig et al. (2003). Since patients with suspected HIV infection in this study were tested voluntarily and less often than that in the other prospective study (Robb et al., 2016), the actual seroconversion time might be even shorter than the reported results. The difference between our results and those of Fiebig et al. (2003) may be attributable to the different study population (Taylor et al., 2015; Robb et al., 2016), because the antibody response to the viral protein depends on the genetic background and health status of patients.

In our study, we focused on recent HIV-1 infection and had excluded subjects that had been tested several times but had not seroconverted during the study period. Both acute/early HIV-1 infection and late-stage HIV infection would have been tested as HIV-1 antibodies negative or indeterminate. However, recent/early HIV-1 infection is more common than non-symptomatic late-stage HIV infection, and these patients are likely to seroconvert on follow-up within weeks (Hecht et al., 2011; Centers for Disease Control [CDC], 2014). Detection of early HIV-1 infection is a key imperative both from a therapeutic as well as a preventive perspective; studies have shown that intervention during early HIV-1 infection can reduce the size of the HIV-1 reservoir (Hill et al., 2014) and help minimize the epidemic spread (Hecht et al., 2011; Cohen et al., 2013; Robb et al., 2016). In our previous study (Liu et al., 2016), newly infected individuals with negative WB result showed higher HIV-1 loads than those with indeterminate WB; however, these were not flagged for re-testing after 4 weeks based on the current HIV testing algorithm in China. Accurate estimation of seroconversion is very important for HIV-1 prevention through universal testing and treatment strategy (Cohen et al., 2011a, 2012; Powers et al., 2011; Robb et al., 2016).

The pattern of WB bands has been used as a serologic marker of recent HIV-1 seroconversion (Sudha et al., 2006; Schupbach et al., 2007, 2013); in addition, the number, intensity, and order of emergence of WB bands are associated with the staging of HIV-1 infection (Tuaillon et al., 2017; Huang et al., 2018). In a previous study by the Acute Infection and Early Disease Research Program network, p31 antibody was found absent from 98 newly infected HIV patients (Hecht et al., 2002). Based on this finding, p31 was used as a viral marker to distinguish Fiebig stage V, recent HIV-1 infection, from stage VI, the early chronic infection (Fiebig et al., 2003; Cohen et al., 2011b; Tuaillon et al., 2017). In this study, not surprisingly, significant differences were observed between the seroconversion duration of subjects with and without p31 antibody band in their final WB pattern, irrespective of whether their initial WB test result was negative (Figure 2) or indeterminate (Figure 3); these findings provide direct and adequate evidence to support the Fiebig staging. In addition, p31 seroconversion from negative WB to positive WB occurred over a mean duration of 58.11 (95% CI, 44.30–71.92) days, which is much shorter than that in Keating’s study (Keating et al., 2016); this suggests that the lack of p31 band may characterize HIV infection for a period of less than 1 year (Tuaillon et al., 2017). Interestingly, antibody against p66, like p31, showed potential as a viral marker, as there was a significant difference between seroconversion of individuals with and without p66 antibody. Further analysis showed that p66 band had similar window period as that for p31. To the best of our knowledge, this feature of p66 seroconversion has not been reported previously; in addition, it is not yet clear whether the late emergence of p66 band is a common phenomenon in patients from other regions. However, we propose that the end point for Fiebig stage V should be set at about day 70 post-infection (40 days after stage IV), based on the seroconversion duration of viral markers p31 and p66.

As for the other bands, p51, gp41, and p17 only showed differences of seroconversion in a proportion of patients, which supports the previous finding that *pol* antibodies could be predictors of seroconversion (Duri et al., 2011). We did not observe any significant difference of seroconversion between individuals with and without gp120 antibody, which is consistent with a recent report (Huang et al., 2018). Gp160 and p24 bands were not included in the analysis because these are the most popular bands (Sudha et al., 2006) and usually became positive earlier than other bands (Hecht et al., 2011).

In summary, we retrospectively analyzed the seroconversion duration among subjects with recent HIV-1 infection who seroconverted from negative WB or indeterminate WB into positive WB. Our data provide direct evidence for the window period of each HIV-1 antibody and suggest that antibody against p66 (like p31) may also serve as a viral marker for distinguishing Fiebig stage V and VI at day 70 post-infection. Additional studies involving a larger sample size covering multiple geographic and genetic backgrounds are needed to clarify the role of p66 as well as other WB bands in the progression of HIV-1 infection.

## Data Availability

The datasets for this manuscript are not publicly available because the datasets included the patients’ information, which could not be made publicly available online. Requests to access the datasets should be directed to M-QL, liumq33@hotmail.com.

## Ethics Statement

Written informed consent and data pertaining to demographic characteristics were collected at the time of the first HIV test. The study was approved by the Institutional Review Board of Wuhan CDC.

## Author Contributions

M-QL designed the study. W-HK and M-QL wrote the manuscript. PL and M-QL analyzed the data. All authors collected the data and contributed to the writing and proofreading of this manuscript.

## Conflict of Interest Statement

The authors declare that the research was conducted in the absence of any commercial or financial relationships that could be construed as a potential conflict of interest.

## References

[B1] Bottone P. D. Bartlett A. H. (2017). Diagnosing acute HIV Infection. *Pediatr. Ann.* 46 e47–e50. 10.3928/19382359-20170118-01 28192577

[B2] Branson B. M. Stekler J. D. (2012). Detection of acute HIV infection: we can’t close the window. *J. Infect. Dis.* 205 521–524. 10.1093/infdis/jir793 22207652

[B3] Brostrom B. Granich R. Gupta S. Samb B. (2014). Reimagining HIV testing in an era of ART. *AIDS Res. Hum. Retroviruses Suppl.* 1:A87.

[B4] Centers for Disease Control [CDC] (2014). *Laboratory Testing for the Diagnosis of HIV Infection: Updated Recommendations*. Atlanta, GA: Centers for Disease Control and Prevention and Association of Public Health Laboratories.

[B5] Cohen M. S. Chen Y. Q. Mccauley M. Gamble T. Hosseinipour M. C. Kumarasamy N. (2011a). Prevention of HIV-1 infection with early antiretroviral therapy. *N. Engl. J. Med.* 365 493–505. 21767103 10.1056/NEJMoa1105243 PMC3200068

[B6] Cohen M. S. Shaw G. M. Mcmichael A. J. Haynes B. F. (2011b). Acute HIV-1 infection. *N. Engl. J. Med.* 364 1943–1954. 21591946 10.1056/NEJMra1011874 PMC3771113

[B7] Cohen M. S. Dye C. Fraser C. Miller W. C. Powers K. A. Williams B. G. (2012). HIV treatment as prevention: debate and commentary–will early infection compromise treatment-as-prevention strategies? *PLoS Med.* 9:10. 10.1371/journal.pmed.1001232 22802728 PMC3393667

[B8] Cohen M. S. Gay C. L. Busch M. P. Hecht F. M. (2010). The detection of acute HIV infection. *J. Infect. Dis.* 202 S270–S277. 10.1086/655651 20846033

[B9] Cohen M. S. Smith M. K. Muessig K. E. Hallett T. B. Powers K. A. Kashuba A. D. (2013). Antiretroviral treatment of HIV-1 prevents transmission of HIV-1: where do we go from here? *Lancet* 382 1515–1524. 10.1016/S0140-6736(13)61998-4 24152938 PMC3880570

[B10] Duri K. Muller F. Gumbo F. Z. Kurewa N. E. Rusakaniko S. Chirenje M. Z. (2011). Human Immunodeficiency Virus (HIV) types Western blot (WB) band profiles as potential surrogate markers of HIV disease progression and predictors of vertical transmission in a cohort of infected but antiretroviral therapy naive pregnant women in Harare. Zimbabwe. *BMC Infect. Dis.* 11:7. 10.1186/1471-2334-11-7 21211021 PMC3022718

[B11] Fiebig E. W. Wright D. J. Rawal B. D. Garrett P. E. Schumacher R. T. Peddada L. (2003). Dynamics of HIV viremia and antibody seroconversion in plasma donors: implications for diagnosis and staging of primary HIV infection. *AIDS* 17 1871–1879. 10.1097/00002030-200309050-00005 12960819

[B12] Garland F. C. Garland C. F. Gorham E. D. Brodine S. K. (1996). Western blot banding patterns of HIV rapid progressors in the U.S. Navy seropositive cohort: implications for vaccine development. navy retroviral working group. *Ann. Epidemiol.* 6 341–347. 10.1016/s1047-2797(96)00053-1 8876845

[B13] Hecht F. M. Holte S. Busch M. P. Hogan C. Little S. Schacker T. (2002). “Absence of p31 band identifies persons with recent HIV seroconversion,” in *Proceedings of the XIVth International AIDS Conference*, Barcelona.

[B14] Hecht F. M. Wellman R. Busch M. P. Pilcher C. D. Norris P. J. Margolick J. B. (2011). Identifying the early post-HIV antibody seroconversion period. *J. Infect. Dis.* 204 526–533. 10.1093/infdis/jir304 21791654 PMC3144168

[B15] Hill A. L. Rosenbloom D. I. Fu F. Nowak M. A. Siliciano R. F. (2014). Predicting the outcomes of treatment to eradicate the latent reservoir for HIV-1. *Proc. Natl. Acad. Sci. U.S.A.* 111 13475–13480. 10.1073/pnas.1406663111 25097264 PMC4169952

[B16] Huang J. Wang M. Huang C. Liang B. Jiang J. Ning C. (2018). Western blot-based logistic regression model for the identification of recent HIV-1 infection: a promising HIV-1 surveillance approach for resource-limited regions. *Biomed. Res. Int.* 2018:4390318. 10.1155/2018/4390318 29568753 PMC5820577

[B17] Keating S. M. Kassanjee R. Lebedeva M. Facente S. N. Macarthur J. C. Grebe E. (2016). Performance of the bio-rad geenius HIV1/2 supplemental assay in detecting “recent” HIV infection and calculating population incidence. *J. Acquir. Immune Defic. Syndr.* 73 581–588. 10.1097/qai.0000000000001146 27509247 PMC5110377

[B18] Kondo M. Sudo K. Sano T. Kawahata T. Itoda I. Iwamuro S. (2018). Comparative evaluation of the geenius HIV 1/2 confirmatory assay and the HIV-1 and HIV-2 western blots in the Japanese population. *PLoS One* 13:e0198924. 10.1371/journal.pone.0198924 30379808 PMC6209130

[B19] Linley L. Ethridge S. F. Oraka E. Owen S. M. Wesolowski L. G. Wroblewski K. (2013). Evaluation of supplemental testing with the multispot HIV-1/HIV-2 rapid test and APTIMA HIV-1 RNA qualitative assay to resolve specimens with indeterminate or negative HIV-1 Western blots. *J. Clin. Virol.* 58 e108–e112. 10.1016/j.jcv.2013.09.021 24342469

[B20] Liu M. Q. Zhu Z. R. Kong W. H. Tang L. Peng J. S. Wang X. (2016). High rate of missed HIV infections in individuals with indeterminate or negative HIV western blots based on current HIV testing algorithm in China. *J. Med. Virol.* 88 1462–1466. 10.1002/jmv.24490 26856240

[B21] Moon H. W. Huh H. J. Oh G. Y. Lee S. G. Lee A. Yun Y. M. (2015). Evaluation of the bio-rad geenius HIV 1/2 confirmation assay as an alternative to western blot in the korean population: a multi-center study. *PLoS One* 10:e0139169. 10.1371/journal.pone.0139169 26422281 PMC4589337

[B22] Ning T. L. Zheng M. N. Li L. Bai J. Y. Zhao X. Guo Y. (2018). Study on acute HIV-1 infection in men who have sex with men in Tianjin. *Zhonghua Liu Xing Bing Xue Za Zhi* 39 1472–1476. 10.3760/cma.j.issn.0254-6450.2018.11.010 30462956

[B23] Patel P. Bennett B. Sullivan T. Parker M. M. Heffelfinger J. D. Sullivan P. S. (2012). Rapid HIV screening: missed opportunities for HIV diagnosis and prevention. *J. Clin. Virol.* 54 42–47. 10.1016/j.jcv.2012.01.022 22381919 PMC6195213

[B24] Pilcher C. D. Christopoulos K. A. Golden M. (2010). Public health rationale for rapid nucleic acid or p24 antigen tests for HIV. *J. Infect. Dis.* 201(Suppl. 1), S7–S15. 10.1086/650393 20225950

[B25] Powers K. A. Ghani A. C. Miller W. C. Hoffman I. F. Pettifor A. E. Kamanga G. (2011). The role of acute and early HIV infection in the spread of HIV and implications for transmission prevention strategies in Lilongwe. Malawi: a modelling study. *Lancet* 378 256–268. 10.1016/S0140-6736(11)60842-8 21684591 PMC3274419

[B26] Robb M. L. Eller L. A. Kibuuka H. Rono K. Maganga L. Nitayaphan S. (2016). Prospective study of acute HIV-1 infection in adults in East Africa and Thailand. *N. Engl. J. Med.* 374 2120–2130. 10.1056/NEJMoa1508952 27192360 PMC5111628

[B27] Schupbach J. Bisset L. R. Gebhardt M. D. Regenass S. Burgisser P. Gorgievski M. (2011). Diagnostic performance of line-immunoassay based algorithms for incident HIV-1 infection. *BMC Infect. Dis.* 12:88. 10.1186/1471-2334-12-88 22497961 PMC3362747

[B28] Schupbach J. Bisset L. R. Gebhardt M. D. Regenass S. Burgisser P. Gorgievski M. (2012). Diagnostic performance of line-immunoassay based algorithms for incident HIV-1 infection. *BMC Infect. Dis.* 12:88. 10.1186/1471-2334-12-88 22497961 PMC3362747

[B29] Schupbach J. Gebhardt M. D. Scherrer A. U. Bisset L. R. Niederhauser C. Regenass S. (2013). Simple estimation of incident HIV infection rates in notification cohorts based on window periods of algorithms for evaluation of line-immunoassay result patterns. *PLoS One* 8:e71662. 10.1371/journal.pone.0071662 23990968 PMC3753319

[B30] Schupbach J. Gebhardt M. D. Tomasik Z. Niederhauser C. Yerly S. Burgisser P. (2007). Assessment of recent HIV-1 infection by a line immunoassay for HIV-1/2 confirmation. *PLoS Med.* 4:e343. 10.1371/journal.pmed.0040343. 18052604 PMC2100138

[B31] Shao Y. M. Kang L. Y. Wang N. Zhang F. J. Li T. S. Shang H. (2008). *Diagnostic Criteria for HIV/AIDS WS 293-2008*. Beijing: People’s Medical Publishing House.

[B32] Sudha T. Lakshmi V. Teja V. D. (2006). Western blot profile in HIV infection. *Ind. J. Dermatol. Venereol. Leprol.* 72 357–360. 10.4103/0378-6323.27752 17050930

[B33] Tang J. W. Wong B. C. Lam E. Tai V. Lee N. Cockram C. S. (2008). Failure to confirm HIV infection in two end-stage HIV/AIDS patients using a popular commercial line immunoassay. *J. Med. Virol.* 80 1515–1522. 10.1002/jmv.21248 18649337

[B34] Taylor D. Durigon M. Davis H. Archibald C. Konrad B. Coombs D. (2015). Probability of a false-negative HIV antibody test result during the window period: a tool for pre- and post-test counselling. *Int. J. STD AIDS* 26 215–224. 10.1177/0956462414542987 25033879

[B35] Toussova O. V. Kozlov A. P. Verevochkin S. V. Lancaster K. E. Shaboltas A. V. Masharsky A. (2018). A cohort approach to real-time detection of acute hiv infections among people who inject drugs in St. Petersburg, Russia. *AIDS Res. Hum. Retrovirus.* 34 261–268. 10.1089/AID.2017.0076 29145741 PMC5863101

[B36] Tuaillon E. Sanosyan A. Pisoni A. Liscouet J. Makinson A. Perre P. V. (2017). Staging of recent HIV-1 infection using geenius rapid confirmatory assay compared to INNO-LIA, new lav and Blot 2.2 *assays*. *J. Clin. Virol.* 95 47–51. 10.1016/j.jcv.2017.08.003 28843384

[B37] Wang J. B. Zhang N. Yu H. Y. Li Y. L. Duan X. Yan H. (2013). Study on the role of western blot band profile for the detection of recent HIV infection. *Zhonghua Liu Xing Bing Xue Za Zhi* 34 998–1002. 24377995

[B38] World Health Organization [WHO] (2017). Summary of the Global HIV Epidemic (2017) [Online]. Available: http://www.who.int/hiv/data/2017_summary-global-hiv-epidemic.png?ua=1 (accessed July, 2018).

